# Preharvest and postharvest techniques that optimize the shelf life of fresh basil (*Ocimum basilicum* L.): a review

**DOI:** 10.3389/fpls.2023.1237577

**Published:** 2023-09-08

**Authors:** Lara J. Brindisi, James E. Simon

**Affiliations:** New Use Agriculture and Natural Plant Products Program, Department of Plant Biology and the Center for Agricultural Food Ecosystems (RUCAFE), Rutgers University, New Brunswick, NJ, United States

**Keywords:** storage, shipping, handling, cold stress, chilling injury

## Abstract

Basil (*Ocimum basilicum* L.) is a popular specialty crop known for its use as a culinary herb and medicinal plant around the world. However, its profitability and availability are limited by a short postharvest shelf life due to poor handling, cold sensitivity and microbial contamination. Here, we comprehensively review the research on pre- and postharvest techniques that extend the shelf life of basil to serve as a practical tool for growers, distributors, retailers and scientists. Modifications to postharvest storage conditions, pre- and postharvest treatments, harvest time and preharvest production methods have been found to directly impact the quality of basil and its shelf life. The most effective strategies for extending the shelf life and improving the quality of basil are discussed and promising strategies that research and industry employ are identified.

## 1 Introduction

Basil (*Ocimum* spp.) is a profitable culinary herb and medicinal plant grown around the world (Simon et al., 1990a; Simon, 1998). It has many applications due to its range of desirable aromas and health benefits as a fresh, dried, frozen or essential oil product in the food, perfume, pharmaceutical and flavoring industries (Simon et al., 1990b; Ch et al., 2015; Shahrajabian et al., 2020; Dhama et al., 2021). Basil is a member of the Lamiaceae, formerly Labiatae, family that includes many other well-known aromatic culinary crops such as mint (*Mentha* spp.), oregano (*Origanum vulgare*), rosemary (*Salvia rosmarinus*, previously *Rosmarinus officinalis*), sage (*Salvia officinalis*) and thyme (*Thymus vulgaris*). There are several important species in the basil family, namely sweet or Italian basil, purple basil, Thai basil (*O. basilicum*), holy or Tulsi basil (*O. tenuiflorum*), lemon basil (*O.* × *africanum*, previously known as *O.* × *citriodorum*) and perennial or clove basil (*O. gratissimum*) (Simon, 1998; Carović-Stanko et al., 2009). Sweet basil is the most common and popular type of basil grown in the U.S. with its Genovese-style aroma formed by a unique bouquet of volatile compounds, although a large diversity of other aromas exist within the genus including cinnamon, lemon, camphor, licorice, rose-like, clove-like and more (Simon et al., 1990a; Patel et al., 2021). The vast majority of the crop is sold as fresh-cut leaves, live rooted plants or cuttings where preservation of freshness, texture, shelf life and aroma are essential.

**Figure 1:**
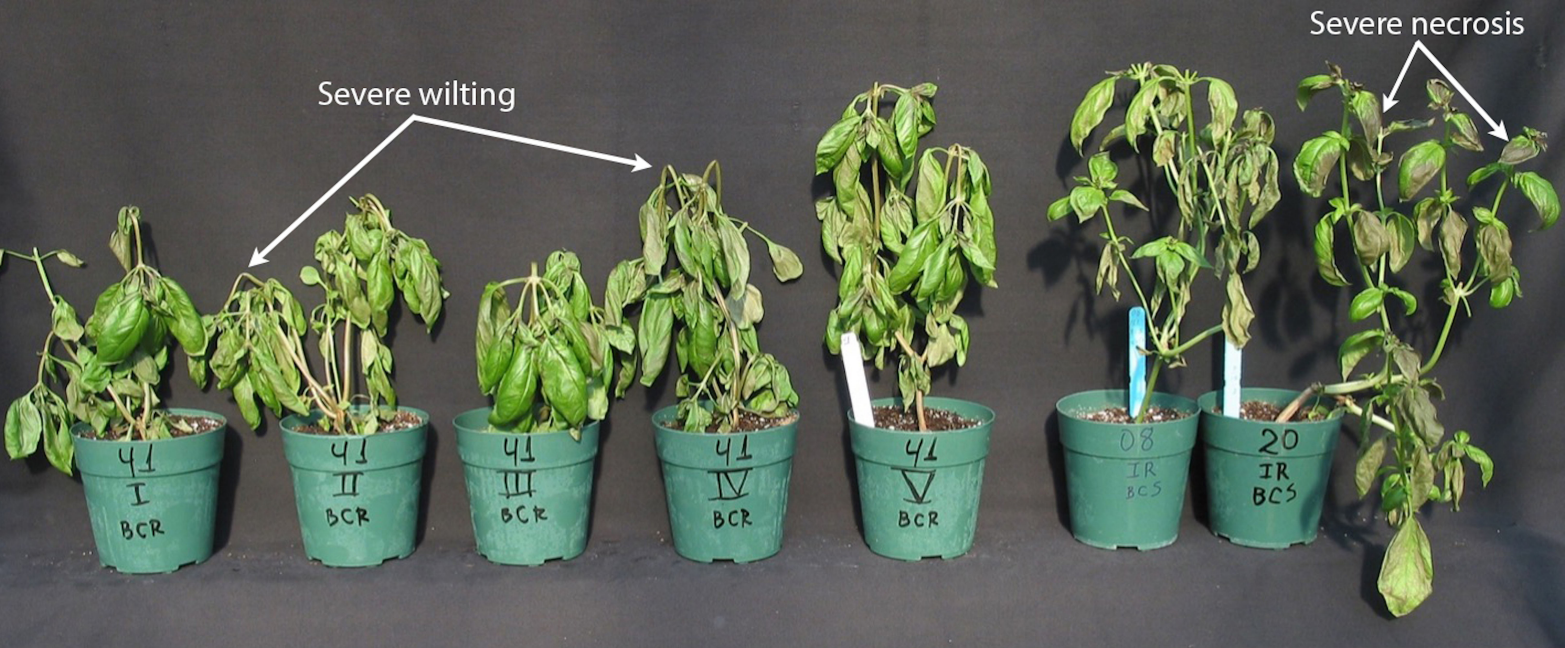
Chilling injury symptoms in sweet basil. Selections of ‘Italian Large-Leaf’ basil showcase different degrees of damage after exposure to chilling temperatures under field conditions. The five plants on the left exhibit wilting severe enough to cause bending at the stem and the two plants on the right exhibit the most severe leaf blackening or necrosis of those depicted. Reproduced with permission (Ribeiro and Simon, 2007).

Sweet basil is highly sensitive to cold temperatures, which is widespread amongst plants with tropical origins (Morris, 1982; Lange and Cameron, 1994; Paton et al., 2004). Storage at less than 12°C (54°F) causes injury in basil leaves and temperatures less than 10°C (50°F) induces severe if not total injury (Cantwell and Reid, 1993; Lange and Cameron, 1994; Harley et al., 2004; Paton et al., 2004). Chilling injury symptoms include leaf necrosis, which appears as leaf spotting or browning, wilting or loss of leaf turgidity and decay (Lange and Cameron, 1994; Ribeiro and Simon, 2007; Brindisi et al., 2021). Leaves are rendered inedible when cases are moderate to severe, as shown in **Figure 1** (Ribeiro and Simon, 2007). Chilling sensitivity in basil is a major problem for growers, distributers and retailers, because basil and other herbs are often stored and shipped at low temperatures with other fresh produce to minimize disease and decay (da Silva et al., 2005; Aharoni et al., 2010; Delbeke et al., 2015; Tirawat et al., 2016; Valiolahi et al., 2019). Thus, special handling for this crop is highly necessary.

Sweet basil has a relatively short shelf life as a fresh herb even when compared to other types of basil (Cantwell and Reid, 1993; Ciriello et al., 2023). Fresh cuttings of sweet basil have been reported to remain marketable from only 2 to 14 days or an average of 6.8-7.3 days at 20-25°C, which is considered room temperature (Lange and Cameron, 1994). Such a short shelf life would preclude the use of conventional storage and distribution value chains. Storage life improves at a slightly lower storage temperature of 15°C with sweet basil lasting an average of 12.5 days. However, the shelf life of basil has been shown to sharply decline at colder temperatures, lasting an average of only 8.3 days, 3.2 days and 1.6 days at 10°C, 5°C and 0°C, respectively. Other herbs tend to have the inverse relationship between shelf life and storage temperature. For instance, watercress (*Nasturtium officinale*) and mint can maintain freshness at 0°C for up to 4 weeks in bags and only 2-4 days at 20°C (Hruschka and Wang, 1979). Parsley (*Petroselinum crispum*) maintains marketability for an additional 21-36 days at 0°C in bags and only 3 days at 20°C after 4 weeks of storage in crates with top ice.

Shelf life here is defined as the length of time until the first obvious signs of deterioration appear on the stored leaves either due to fungal growth or chilling injury. Yet, the definition of shelf life varies in the literature. An example of a more ‘lenient’ description of shelf life is when the limit of marketability is defined as black stains appearing on more than 30% of the leaf area and the limit of edibility is defined as black stains appearing on 30-50% of the leaf area (Changsawake et al., 2015; Changsawake et al., 2017). More lenient descriptions of shelf life such as this result in longer than expected storage times.

Shelf life is measured through visual, chemical or physiological assessments. Visual assessment of leaf necrosis is the most common method for assessing the degree of chilling injury because it directly translates to the marketability of the end product in which moderate to severe leaf browning is considered unmarketable (Lange and Cameron, 1994; Brindisi et al., 2021). Several chemical and physiological responses in plants correlate with chilling stress and are evaluated to hypothesize the mechanisms eliciting chilling tolerance after exposure to different treatments or conditions. For example, sweet basil leaves with visibly less injury under cold stress increase in soluble sugars, starch and antioxidants, maintain higher levels of chlorophyll, protein compounds and leaf water content, produce less ethylene and decrease in rates of electrolyte leakage (Ribeiro and Simon, 2007; Hassan and Mahfouz, 2010; Ghasemzadeh et al., 2016; Sharma et al., 2018; Larsen et al., 2022a). Visual assessments are also useful when observing the development of molds or yeasts, though microbiological analyses are more suitable for the quantification of microbial growth and early detection of fungi and bacteria (da Silva et al., 2005; Delbeke et al., 2015; Tirawat et al., 2016; Valiolahi et al., 2019).

Aroma and taste profiling are important factors when assessing shelf life because they are main drivers for consumer acceptance. The major aromatic or volatile compounds produced by sweet basil are linalool, eucalyptol (also known as 1,8-cineole), methyl chavicol (also known as estragole) and eugenol and are responsible for floral, fresh, licorice and clove aromas, respectively (Lee et al., 2005; Patel et al., 2021). Volatile compounds generally decrease with increased storage time, especially at low temperatures of 4°C (Cozzolino et al., 2016; Fratianni et al., 2017). Preservation of such aromatic compounds is crucial during postharvest handling to ensure that the basil remains appealing to consumers after shipping and storage. While taste and flavor are also determinants of quality, studies to date have focused on aroma volatiles largely due to the extensive body of literature and ease and familiarity of examining essential oils. Furthermore, lexicons have been developed to evaluate basil aroma (Lee et al., 2017; Patel et al., 2021), but a lexicon for taste and flavor has not yet been reported.

**Figure 2:**
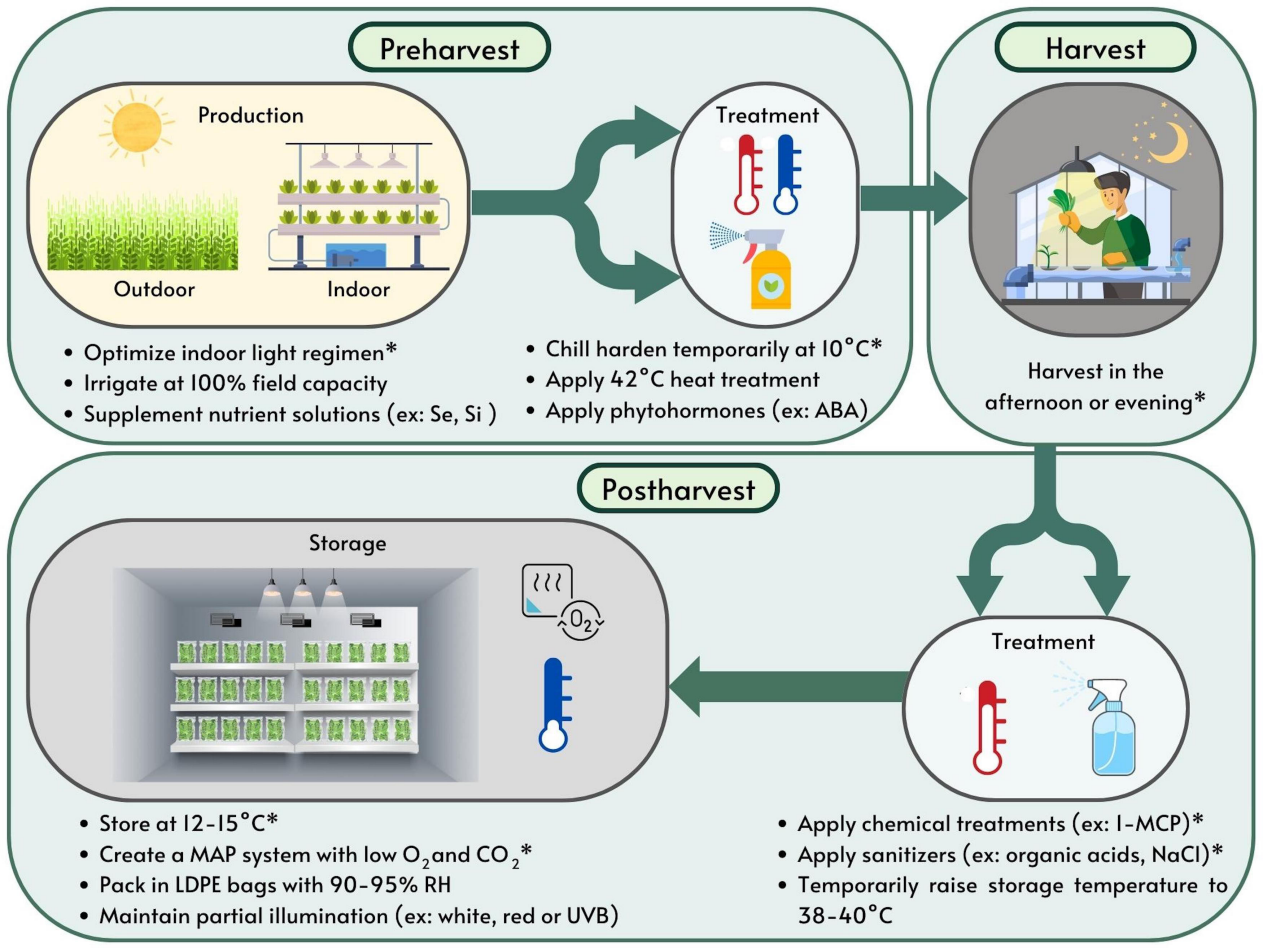
Summary of pre- and postharvest techniques now practiced for optimizing shelf-life in sweet basil. *Represents the most effective strategies for optimizing shelf-life researched and reported in the literature to date. ABA, abscisic acid; H_2_O_2_, hydrogen peroxide; LDPE, low density polyethylene; MAP, modified atmospheres packaging; NaCl, sodium chloride; RH, relative humidity.

Several strategies have been researched to extend the shelf life of basil. Storage conditions, pre- and postharvest treatments, production methods and harvesting time all impact the shelf life and quality of harvested fresh basil as visually depicted in **Figure 2** and summarized in **Table 1**. While genetic background also influences chilling response in basil, little is known about the regulation of cold responsive gene expression in basil and no commercial sweet basils with confirmed chilling tolerance have been released to date (Ribeiro and Simon, 2007; Zhan et al., 2016). Hence, there is great benefit in adopting measures that optimize the postharvest shelf life of basil. This review seeks to summarize the existing literature to identify best practices for obtaining high-quality sweet basil and extending shelf life.

**Table 1:** Pre- and postharvest techniques with beneficial effects on basil postharvest quality.

Category	Type of strategy	Significant strategies	Stage	Storage Temperature	Reported effects in basil
Storage Conditions	Storage temperature	Store harvested leaves under mild refrigeration	Postharvest	12-15°C	Minimize chilling injury (Lange and Cameron, 1994; Lange and Cameron, 1997; Meir et al., 1997; Rodeo and Mitcham, 2023), microbial growth (da Silva et al., 2005; Delbeke et al., 2015) and loss of volatile aroma compounds, polyphenols and antioxidants (Sharma et al., 2005; López-Blancas et al., 2014; Cozzolino et al., 2016; Ghasemzadeh et al., 2016; Fratianni et al., 2017; Rodeo and Mitcham, 2023)
	Artificial lighting	Store under illumination	Postharvest	12°C	Maintain more herb aroma (Anderson et al., 2011)
	Artificial lighting	Apply low intensity white or red light pulses (30–37 µmol·m−2s−1) for 2 h daily during storage	Postharvest	20°C	Retain chlorophyll and protein content, minimize ammonium accumulation and increase soluble sugar content in the leaves (Liu et al., 2015)
	Artificial lighting	Add UV-B light (3.60 W·m−2) for 8 h during storage	Postharvest	Unspecified	Preserve secondary metabolites and antiproliferative activity, though only observed after 24 h (Ghasemzadeh et al., 2016)
	Controlled atmosphere	Maintain 1.5-2% O_2_ and 0% CO_2_ in N_2_ during storage	Postharvest	12°C and 20°C	Extend shelf life by up to 27 days (Lange and Cameron, 1998; Amodio et al., 2005; Anderson et al., 2011)
	Controlled atmosphere	Add 5% CO_2_ to 16% O_2_ during storage	Postharvest	5°C	Ameliorate chilling injury after 3 days of storage (Rodeo and Mitcham, 2023)
	Controlled atmosphere	Add 2.5 kPA CO_2_ to air during storage	Postharvest	12°C	Inhibit leaf abscission, decay and browning (Kenigsbuch et al., 2015)
	Packaging	Store basil in low density polyethylene bags	Postharvest	10-18°C	Minimize fresh weight loss, improve visual quality (Aharoni et al., 1993) and preserve chlorophyll content, total phenols, total flavonoids, antioxidant and cell membrane (Sharma et al., 2018)
	Humidity	Store basil at >90% RH	Postharvest	5°C and 12°C	Maintain leaf quality and slow enzymatic activity (Sharma et al., 2018)
Treatments	Cold acclimation	Chill harden at 10°C for 4 h 1 day prior to storage	Preharvest	5°C	Extend shelf-life by 5 days (Lange and Cameron, 1997)
	Heat treatment	Heat at 38-40°C for 8 h after harvest	Postharvest	9°C and 12°C	Reduce cold injury and inhibit leaf decay and gray mold (Aharoni et al., 2010)
	Heat treatment	Heat at 42°C for 2-3 days prior to harvest	Preharvest	Unspecified	Eliminate postharvest leaf spot (Kenigsbuch et al., 2010)
	Phytohormone application	Apply ABA (1000-1500 mg·L−1) as a foliar treatment 24 h prior to harvest	Preharvest	3.5°C	Reduce ion leakage (Satpute et al., 2019)
	1-MCP application	Treat with single dose of 1-MCP at 0.4 g·m−3 applied for 8 h or 0.3 cm3·m−3 applied for 24 h at 15°C in the dark	Postharvest	20°C	Extend shelf-life 4-6 days and improve freshness or turgor and yellowing or browning (Hassan and Mahfouz, 2010; Patiño et al., 2018)
	Sanitizer	Dip leaves in 5-20 ppm of electrolyzed water for 30 min	Postharvest	20-25°C	Increase the shelf-life of basil by retaining higher concentrations of chlorophyll and carotenoids (Wilson et al., 2019)
	Sanitizer	Immerse leaves in 2% lactic acid and 2% H_2_O_2_ heated to 40°C	Postharvest	4°C	Reduce microbial counts and maintain consumer acceptance for appearance and aroma (Valiolahi et al., 2019)
	Fumigation	Treat basil with nitric oxide (1-5 ul·L-1) for 2 h at 20°C	Postharvest	5°C and 20°C	Reduce water loss by 8% after 24 h storage (Ku et al., 2015)
	Vapor treatment	Fumigate basil with vinegar and 4% acetic acid for 10 m	Postharvest	12°C	Extend shelf life from 17.8 to 25.4 d, minimize fresh weight loss, slow chlorophyll degradation, elevate radical scavenging and lessen membrane leakage and lipid peroxidation (Changsawake et al., 2017; Teerarak et al., 2020)
	Vapor treatment	Apply vapor treatments of tea tree oil (1 mL·L-1) for 1 min at 16°C	Postharvest	16°C	Extend shelf-life from 4.7 to 10.3 d, enhance freshness, maintain volatile oil content, slow chlorophyll degradation and maximize antioxidant peroxidase activity (Fetouh, 2015)
	Nanotechnology	Apply thyme volatile oil loaded chitosan nanoparticles to leaves	Postharvest	20°C	Extend shelf-life by 2.4-fold, minimize weight loss, reduce malondialdehyde and hydrogen peroxide and retain higher levels of chlorophyll, carotenoids and volatile oils (Hassan et al., 2021)
	Nanotechnology	Apply nano-curcumin and nano-rosmarinic acid as foliar treatments (60-90 µg)	Postharvest	10°C	Reduce fresh weight loss, slow the degradation of chlorophyll, retain volatile oil content and inhibit microorganism growth (Hammam and Shoala, 2020)
Production methods	Artificial lighting	Use 80:20 ratio of red and green light for indoor cultivation	Preharvest	6-20°C	Slightly increase chilling tolerance and shelf-life (Jensen et al., 2018)
	Artificial lighting	Supplement the last 3 weeks of the growth cycle (10 DAS) with far-red light (180 μmol·m-2·s-1)	Preharvest	4°C and 12°C	Improve overall visual quality by 50% and increase plant height, leaf area, percentage of plant dry matter content, plant fresh mass and chilling tolerance (Larsen et al., 2020; Larsen et al., 2023)
	Artificial lighting	Increase light intensity (300-600 μmol·m−2·s−1) for the last 5 days of the production cycle	Preharvest	4°C and 12°C	Increase leaf resiliency, plant fresh mass, plant dry matter content, visual quality, and levels of soluble sugars, starch and nonenzymatic antioxidants, but does not improve chilling injury symptoms (Larsen et al., 2020; Larsen et al., 2022b; Larsen et al., 2023)
	Artificial lighting	Supplement with UV-B light at 0.5 kg·s−3 for 4 days and then white light for 1 day prior to harvest	Preharvest	10°C	Maintain higher levels of phenolic acids and antioxidant activity (Dos et al., 2020)
	Drought stress	Irrigate at 100% field capacity	Preharvest	12°C	Maintain higher levels of volatile oils (Jordan et al., 2017)
	Plant nutrition	Supplement nutrient solution with 4 mg·L−1 Se	Preharvest	8°C	Reduce ethylene production and increase antioxidants, total phenols and rosmarinic acid (Puccinelli et al., 2020)
	Plant nutrition	Supplement nutrient solution with silicon 75 ppm Si	Preharvest	Unspecified	Increase plant height and weight, potentially increase frost tolerance (Li et al., 2020)
	Time of harvest	Harvest basil in the afternoon or evening	NA	12°C	Extend shelf-life two-fold (Lange and Cameron, 1994) and significantly improve cold tolerance (Aharoni et al., 2010)

1-MCP, 1-methylcyclopropene; ABA, abscisic acid; CO_2_, carbon dioxide; DAS, days after sowing; H_2_O_2_, hydrogen peroxide; N_2_, nitrogen; O_2_, oxygen; RH, relative humidity; Se, selenium; Si, silicon; UV, ultraviolet.

## 2 Storage conditions

### 2.1 Storage temperature

Fresh basil maintains the longest shelf life when stored at refrigeration temperatures of 12-15°C to avoid chilling injury and uphold aroma profile, fresh weight and greenness. Most other herbs preserve freshness and visual quality best when stored at 0°C or slightly above freezing (Aharoni et al., 1993; Cantwell and Reid, 1993; Eskin and Hoehn, 2013). However, severe chilling injury occurs in basil at storage temperatures of 5°C and below. Basil leaves blacken from necrosis in as little as 1 day at 0°C or 3 days at 5°C (Lange and Cameron, 1994; Lange and Cameron, 1997; Meir et al., 1997; Brindisi et al., 2021; Rodeo and Mitcham, 2023). Moderate chilling injury occurs at 7.5-10°C and ceases at temperatures of 12°C and greater (Lange and Cameron, 1994; Meir et al., 1997; López-Blancas et al., 2014). Lower storage temperatures are also detrimental for retaining most sweet basil secondary metabolites responsible for aroma, pigmentation and pharmacological and antioxidant activity (Luna et al., 2015; Cozzolino et al., 2016; Ghasemzadeh et al., 2016; Fratianni et al., 2017). Basil samples stored for 9 days at 4°C were significantly lower in volatile aroma compounds, polyphenols and antioxidants than those stored at 12°C (Sharma et al., 2005; Cozzolino et al., 2016; Fratianni et al., 2017). Sudden declines in basil volatile compounds have been observed even earlier after only 3 days at 5°C (Rodeo and Mitcham, 2023). While some secondary metabolites such as carotenoids and a handful of volatile compounds were better conserved at 4-5°C compared to 12-20°C, relatively higher storage temperatures are favorable for the major volatiles responsible for aroma in basil (López-Blancas et al., 2014; Cozzolino et al., 2016; Rodeo and Mitcham, 2023). Contrary to preserving leaf freshness and aroma, storing basil at cooler temperatures of <15°C is favorable for inhibiting fungi and bacteria (da Silva et al., 2005; Delbeke et al., 2015). Fungal molds developed on basil after only 3 days of storage at 22°C and 5 days at 15°C but were not reported to grow during storage of 7 days at 7°C (Delbeke et al., 2015). Similarly, storage at 10°C reduced microbial counts on basil compared to 20°C (da Silva et al., 2005). *Salmonella typhimurium* and *Escherichia coli* counts were also lower at colder temperatures throughout storage of fresh basil leaves (Delbeke et al., 2015). A moderate refrigeration temperature of 12-15°C during storage reconciles the challenge of simultaneously managing chilling injury, aroma preservation and microbial growth in basil.

### 2.2 Artificial lighting

Basil may benefit from partial illumination during storage. Exposure to extended periods of darkness is known to induce leaf senescence in some plant species, especially for detached leaves (Quirino et al., 2000). Storing basil under continuous fluorescent light (4.6 m^–1^·s^–1^) instead of in the dark at 12°C locally improved the maintenance of herb aroma (Anderson et al., 2011). This result was similar in rosemary when stored at 1°C. Yet, continuous lighting is known to accelerate physiological activity, chlorophyll loss and transpiration in plants (Olarte et al., 2009; Sanz et al., 2009). Asparagus (*Asparagus officinalis*) stored at 4°C under cool white, green (485-495 and 540-555 nm), red (580-630 nm) and blue (400-550) light all displayed increased physiological activity, which accelerated the mechanisms involved in tissue degradation and decreased shelf life (Sanz et al., 2009). Maintaining a cycle of light and dark periods during storage extends shelf life more than constant light or darkness in some leafy green crops. Leaf discs from kale (*Brassica oleracea* cv. acephala group), cabbage (*Brassica oleracea*), green leaf lettuce (*Lactuca sativa*) and spinach (*Spinacia oleracea*) had superior tissue integrity, green coloration and chlorophyll content after storage under 12 h light followed by 12 h dark compared to 24 h light or 24 h dark (Liu et al., 2015).

Supplying low intensity white or red light pulses or UV-B light during postharvest storage may also help preserve basil quality during storage. Light pulses can be used to disrupt the darkness without the potential adverse effects of continuous light in a cost-effective manner. Low intensity white, red or far-red light pulses at 30–37 µmol·m^−2^s^−1^ were delivered to harvested basil leaves for 2 h daily throughout 5 days of storage at 20°C in the dark (Costa et al., 2013). The white light pulses helped retain chlorophyll and protein content, minimized ammonium accumulation and increased soluble sugar content in the leaves, suggesting the treatment delayed senescence, though a visual assessment was not reported. Basil leaves responded similarly to pulses of red light but not to those of far-red light in terms of chlorophyll, protein and ammonium levels, suggesting that phytochromes mediate low intensity light pulses to delay senescence. UV-B treatment of basil leaves during storage at 3.60 W·m^−2^ for 8 h also suggested potential for preserving secondary metabolites, however, basil plants were only assessed for phenolic content, flavonoids, antioxidants and antiproliferative activity after 24 h of storage at an unknown temperature and results could be significantly different after longer storage times under varying temperatures (Ghasemzadeh et al., 2016).

### 2.3 Controlled atmosphere

Managing low percentages of oxygen (O_2_) and no or low percentages of carbon dioxide (CO_2_) in storage environments can be advantageous for extending the shelf life of fresh basil leaves. Controlled atmosphere in storage occurs when the atmospheric conditions differ from ambient air, which is composed of 78% nitrogen (N_2_), 21% O_2_ and 0.04% CO_2_ (Allen, 2000; Eskin and Hoehn, 2013). Low levels of O_2_ and high levels of CO_2_ slow catabolic processes (i.e., respiration, aging and ethylene activity), and thus extend the storage life of fresh produce (Eskin and Hoehn, 2013). While storage with low levels of O_2_ has been shown to be beneficial for extending the postharvest life of basil, storage with high levels of CO_2_ has caused injury to basil leaves. Basil is relatively sensitive to CO_2_. When basil (*Ocimum basilicum*), coriander (*Coriandrum sativum*), mint (*Mentha spicata*) and parsley (*Petroselinum crispum*) leaves were exposed to 96% CO_2_ for 1 h, all the herbs improved in quality after 10 days of storage except basil, which could not tolerate the treatment (Wilson et al., 2019). An ideal postharvest atmospheric ratio of 1.5% O_2_ + 0% CO_2_ in N_2_ was determined after basil stored in micro-perforated bags yielded the longest shelf life of 45 days compared to 18 days in the dark at 20°C, though no atmospheric ratios alleviated chilling injury at 5°C (Lange and Cameron, 1998). Lower percentages of O_2_ (<1%) formed dark soggy lesions after only 3 days in storage. Higher percentages of CO_2_ minimized shelf life (>5%) and caused interveinal brown spotting in postharvest basil leaves (7.5-10%). Similarly, basil stored in jars with 2% O_2_ + 0% CO_2_ in N_2_ resulted in firmer, greener and less injured basil leaves than those stored with 2% O_2_ + 3% CO_2_ in N_2_ or air at 12°C for 20 days (Amodio et al., 2005). Increasing the percentage of O_2_ to 10% and the percentage of CO_2_ to 15-30% was exceedingly detrimental to basil leaf visual quality, texture and aroma (Anderson et al., 2011). Although 0% CO_2_ is ideal for basil storage in N_2_, slightly raising CO_2_ levels may benefit storage under ambient air. Storage in 5% CO_2_ at 5°C minimized the symptoms of chilling injury for up to 3 days in Genovese sweet basil, though not in lemon basil when compared to 0.04% CO_2_ (Rodeo and Mitcham, 2023). Although the conclusions of this study cannot be certain as they did not standardize O_2_ levels, the results are consistent with a prior study that found adding 2.5 kPa CO_2_ to air effectively inhibited leaf abscission, decay and browning in storage at 12°C for 7 days, especially when combined with a single pre-treatment of 0.7 μmol·L^–1^ 1-methylcyclopropene (1-MCP), which is a synthetic growth regulator used to maintain freshness (Kenigsbuch et al., 2015). CO_2_ may act similarly to 1-MCP in preventing senescence by functioning as an ethylene inhibitor in ambient air.

### 2.4 Packaging

Low density polyethylene bags (LDPE) are favorable for the postharvest storage of fresh basil leaves. LDPE is a cellulose film widely used for the packaging and storage of herbs and spices due to several benefits, including being chemically inert, odor-free, a good moisture barrier, highly permeable to gases, inexpensive and able to seal or shrink with heat (King, 2006). Polyethylene (PE) and polypropylene (PP) are similar, common plastic materials (Patiño et al., 2018). However, in one study, it was observed that basil experienced less weight loss and maintained better visual quality when enclosed in a perforated PE liner compared to a perforated PP liner or a paper-lined carton after storage at 10°C for 4 days and then 18°C for 2 days (Aharoni et al., 1993). These results were similar to other herbs i.e., mint (*Mentha* spp.) and chervil (*Anthriscus cerefolium*), which were stored at 5°C for the first 4 days. Storage of leaves in microperforated plastic packaging was compared to non-perforated plastic packaging filled with air, 2% O_2_ + 15% CO_2_ or 10% O_2_ + 30% CO_2_ to create a modified atmospheres packaging (MAP) system (Anderson et al., 2011). The microperforated packaging without MAP performed better than the non-perforated for basil by minimizing water loss, leaf abscission and decay, likely by reducing the accumulation of CO_2_ and ethylene, although not well enough to delay leaf senescence. Conversely, storing basil leaves in non-perforated LDPE with a MAP system of 10.5% O_2_ and 4.2% CO_2_ doubled the shelf life from 8 to 16 days at 11-12°C compared to storing leaves in macro-perforated LDPE (Patiño et al., 2018). The improved performance of non-perforated LDPE packaging in this experiment is likely due to the lower CO_2_ levels. Shelf life could potentially be extended further by removing CO_2_ from the MAP system (Lange and Cameron, 1998; Amodio et al., 2005). The creation of certain modified atmospheres in LDPE packaging may also enable the storage of basil at cooler temperatures. Storing basil in LDPE bags at 5°C preserved biochemical quality compared to storing basil unpackaged on open trays at 12°C in terms of chlorophyll content, total phenols, total flavonoids, antioxidant and cell membrane preservation, though leaf appearance and aroma were not defined (Sharma et al., 2018).

### 2.5 Humidity

Maintaining a humid environment in postharvest storage helps maintain freshness and prolong shelf life. A relative humidity (RH) of >95% is generally recommended for the storage of most vegetables to uphold texture and prevent water loss (Paull, 1999; Eskin and Hoehn, 2013). The Rutgers New Jersey Agricultural Experiment Station recommends a high RH of 90-95% for the storage of basil (Melendez, 2018). Humidity can be increased through the use of a controlled storage chamber or through film packaging (Aharoni et al., 1993). Fresh basil packaged in LDPE bags were stored at lower temperatures of 5°C and 12°C in 90–95% humidity and compared to those stored at ambient temperatures of 12.2-21°C in 37-98% humidity (Sharma et al., 2018). The combination of lower temperature and higher humidity slowed the enzymatic activity in basil and maintained superior leaf quality after 10 days in storage. The high humidity itself may even enable basil to be stored at lower temperatures by minimizing water loss (Aharoni et al., 1993).

## 3 Treatments

### 3.1 Cold acclimation and heat treatment

Basil preconditioned to moderately cold temperatures delays symptoms of chilling injury through a process known as cold acclimation. Cold acclimation is a common technique for reducing chilling injury in fruits and promoting freezing tolerance in green leafy vegetables as intact plants or harvested produce (Fennell and Li, 1987; Sasaki et al., 1996; Byun et al., 2014; Wang and Zhu, 2017; Zhang et al., 2017). Exposure to temperatures that are cooler than normal conditions but not severe enough to induce damage enables plants to amplify gene expression of cold acclimation pathways, accumulate protective soluble solids, sugars, starches and proline and enhance activities of radical oxygen species (ROS) scavenging enzymes to minimize membrane leakage. In one of the early studies on cold injury in basil, the shelf life at 5°C was extended by 3 days when plants were chill-hardened at 10°C for 4 h daily 2 days prior to harvesting (Lange and Cameron, 1997). No additional benefit was detected by chill-hardening the plants for an increased number of days prior to harvest. Shelf life was extended by 5 days when harvested basil was chill hardened 1 day prior to storage at 5°C and compared to non-chilled controls.

**Figure 3:**
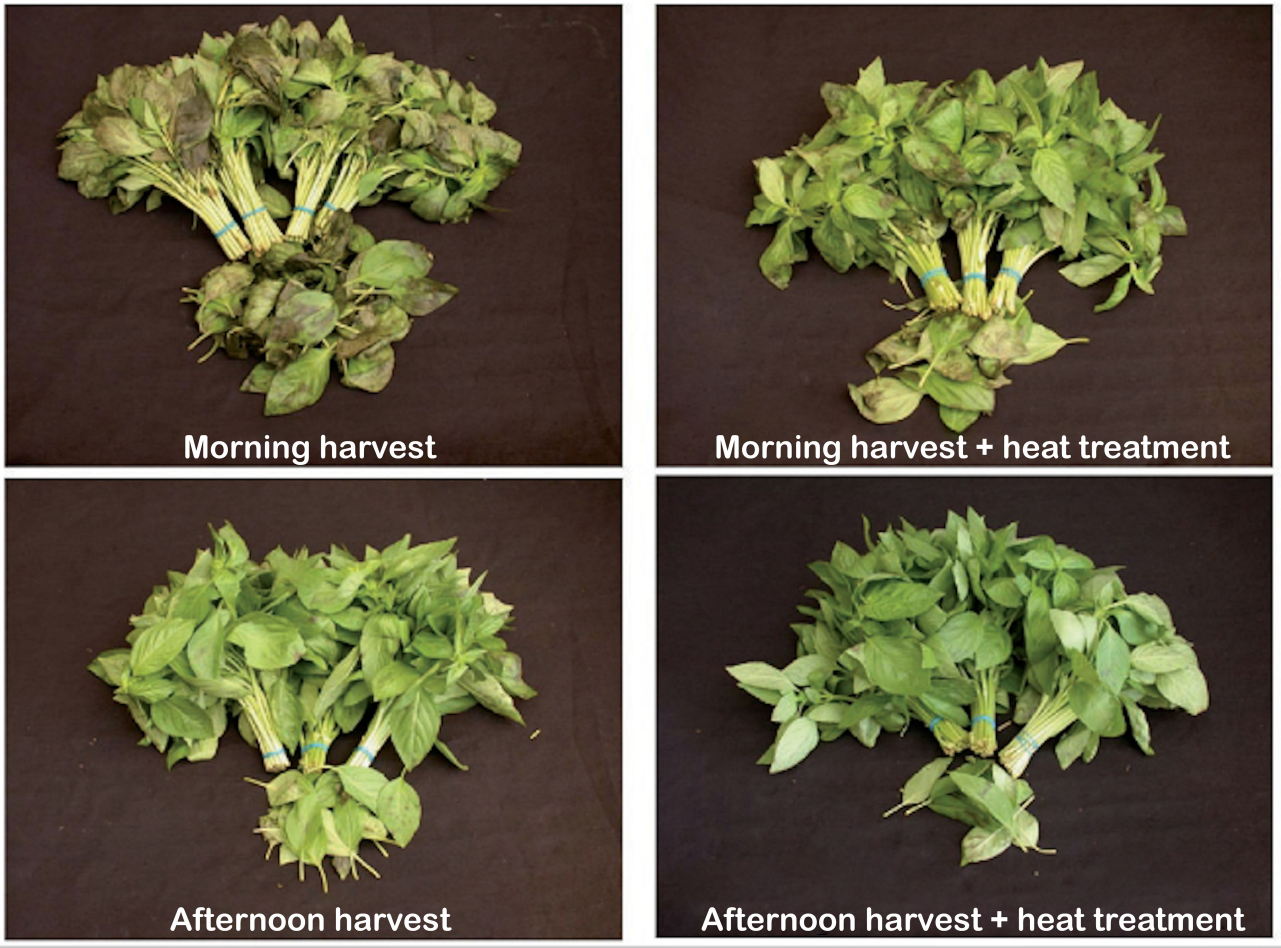
Improved appearance of basil bunches after heat treatment. Basil (‘Perrie’) samples were harvested either in the morning at 6:00 or in the afternoon at 13:00. The harvested samples either remained untreated as controls or received heat treatments at 38-40°C for 4 or 8 h. The samples were then packaged as bunches in polyethylene lined cartons and stored at 10°C for 7 days, and then at 17°C for 2 days. Heat treatments reduced leaf browning, necrosis and abscission, especially for the samples harvested in the morning. Reproduced with permission (Aharoni et al., 2010).

Heat treatments inhibit decay at the pre- and postharvest stages. Postharvest heat treatments encourage chilling tolerance in fruits and vegetables by enhancing heat shock protein gene expression, the ratio of unsaturated fatty acids to saturated fatty acids, the antioxidant system, arginine pathways, sugar metabolism and by altering phenylalanine ammonia-lyase and polyphenol oxidase enzymatic activity (Aghdam and Bodbodak, 2013). Heat treatment of harvested leaves at 38-40°C for 8 h in the afternoon or evening not only reduced basil sensitivity to cold injury at 9°C and 12°C, but also inhibited leaf decay and gray mold attributed to *Botrytis cinerea* as shown in **Figure 3** (Aharoni et al., 2010). Similarly, heat treatment at 42°C for 2-3 days prior to harvest eliminated *Alternaria* growth, which was hypothesized to be the causal agent of black spotting in postharvest basil (Kenigsbuch et al., 2010). Reduction of these postharvest fungal infections is important to maintaining the appearance and freshness of fresh basil leaves throughout storage.

### 3.2 Phytohormone and 1-methylcyclopropene application

Several phytohormones play important roles in regulating plant senescence. Ethylene triggers leaf senescence and promotes leaf abscission by inhibiting auxin synthesis (Iqbal et al., 2017). Cytokinins and gibberellins decrease the biosynthesis of ethylene and inhibit leaf senescence, whereas abscisic acid (ABA) induces senescence. Although ABA can promote leaf aging, it also reduces stomatal conductance, conserving water in plant tissues and alleviating abiotic stresses, such as drought and cold stress (Guo, 2018). Basil leaves grown in the greenhouse and field were less sensitive to chilling injury with decreased ion leakage after 9 days of postharvest storage at 3.5°C when foliar treatments of 1000-1500 mg·L^−1^ ABA were applied to plants 24 h before harvest (Satpute et al., 2019). Other phytohormones have yet to be evaluated on basil for postharvest performance.

Application of 1-MCP is one of the most researched methods for prolonging shelf life for many horticultural crops, including sweet basil, because it slows senescence and leaf abscission by blocking ethylene receptors (Blankenship and Dole, 2003; EPA, 2008). Single dose treatments of 1-MCP at 0.4 g·m^−3^ applied for 8 h and 0.3 cm^3^·m^−3^ applied for 24 h at 15°C on fresh basil leaves extended storage life at 20°C by 4-6 days in terms of freshness or turgor and yellowing or browning (Hassan and Mahfouz, 2010; Patiño et al., 2018). Treatments with 1-MCP minimized loss in fresh weight, slowed the degradation of chlorophyll, retained protein and volatile oil content and greatly reduced leaf abscission during storage of basil leaves and sprigs at 12°C and 20°C likely due to the inhibition of endogenous ethylene production (Aharoni et al., 2010; Hassan and Mahfouz, 2010; Kenigsbuch et al., 2015). Conversely, treatment of 1-MCP at 0.5 g·m^−3^ for 30 s were not significantly different from the control during a 12 day storage period at 5°C or 10°C in terms of postharvest quality, chlorophyll content or fresh weight (Berry et al., 2010). These differences could be due to the deviation in concentration and shorter exposure time compared to the other studies.

### 3.3 Sanitizing washes

Washing produce is an important step in postharvest handling to remove surface contaminants. The addition of chemical sanitizers or disinfectants to washing water is often advised to reduce microbial load and extend shelf life (Joshi et al., 2013). Dipping basil leaves in 5-20 ppm of electrolyzed oxidizing water for 30 min increased the shelf life of basil by retaining higher concentrations of chlorophyll and carotenoids after 14 days of storage at room temperature (Wilson et al., 2019). In a comparison between acidic electrolyzed water treatments (pH=2.5) and 2% lactic acid treatments, the lactic acid immersion was more effective than tap water and acid electrolyzed water at reducing microbial counts of indigenous mesophilic bacteria and pathogenic bacteria such as *E. coli* and *S. typhimurium*, especially when solutions were heated to 50°C (Tirawat et al., 2016). Although, the control treatments of 6% sodium hypochlorite (NaClO) outperformed the lactic acid and electrolyzed water treatments, NaClO may be less desirable as a sanitizer due to formation of byproducts and residues (EPA, 1991; Lee and Huang, 2019). Another comparison between organic acids (i.e., lactic acid, acetic acid and citric acid) alone or in combination with hydrogen peroxide (H_2_O_2_) and sodium chloride (NaCl) as sanitizers on basil identified that a combination of 2% lactic acid and 2% hydrogen peroxide (H_2_O_2_) heated to 40°C performed best at reducing *E. coli* O157:H7 and resulted in the most attractive basil for consumer acceptance in terms of appearance and aroma after 4 days of storage at 4°C (Valiolahi et al., 2019).

### 3.4 Fumigation and vapor treatments

Nitric oxide (NO) fumigation improves the quality of postharvest fruits and vegetables. Proposed mechanisms of action include inhibition of ethylene biosynthesis, enhancement of antioxidant activity, activation of the *CBF* genetic pathway for chilling response and regulation of sugar metabolism (Zhang et al., 2020). A variety of fruits and vegetables were treated with NO gas at 1-500 µL·L^-1^ for 2 h in a nitrogen atmosphere at 20°C and then stored in atmospheric air at 5°C and 20°C (Ku et al., 2015). In all crops, a reduction in water loss was observed after 24 h with NO treatments preventing the most water loss (26%) in Christmas bush (*Ceratopetalum gummiferum* cv. Albery’s Red) and the least water loss (8%) in basil when applied at rates of 1-50 and 1-5 µL·L^-1^, respectively. Fumigation with NO is an effective strategy to prevent postharvest decay in crops for longer durations of storage, such as strawberries for up to 15 days. Harvested strawberry fruits fumigated with only 5-10 ul·L^-1^ NO for 2 h at 20°C in air with 0.1 µL ethylene extended shelf life by >50% (Wills et al., 2000). However, it is unknown how NO fumigation impacts basil shelf life beyond 24 h.

Vapor treatments with vinegar and tea tree oil are effective at extending the shelf life of fresh basil due to their antimicrobial activities. Basil leaves fumigated with upland rice fermented vinegar vapor and 4% acetic acid for 10 minutes lengthened storage in the dark at 12°C from 17.8 to 25.4 days (Changsawake et al., 2017). Basil exposed to 4% acetic acid also lengthened shelf life but only by 16% compared to 35% when in combination with the upland rice fermented vinegar (Teerarak et al., 2020). Both treatments minimized fresh weight loss, slowed the degradation of chlorophyll, elevated radical scavenging and lessened membrane leakage and lipid peroxidation, possibly by reducing the transpiration rate and maintaining cell organelle membrane structure (Changsawake et al., 2017; Teerarak et al., 2020). Tea tree oil distilled from the paper bark tree (*Melaleuca alternifolia* L.) is predominantly comprised of terpinen-4-ol with lesser amounts of γ-terpinene, 1,8-cineole, and *p*-cymene, and is a natural preservative due to its antibacterial, antifungal and antiviral activities (Borotova et al., 2022). Tea tree oil vapor treatments of 1 mL·L^-1^ for 1 min at 16°C extended shelf life of sweet basil leaves from 4.7 to 10.3 days while enhancing freshness, maintaining higher levels of volatile oil content, slowing the degradation of chlorophyll content and maximizing antioxidant enzyme peroxidase activity (Fetouh, 2015). Vaporizing vinegar and essential oils is an attractive sanitizing option that could be acceptable for consumers because it is effective and considered relatively safe and eco-friendly (Falleh et al., 2020). Vapor treatments may also have minimal effect on aroma and taste due to their volatile nature, though the potential impacts on aroma and taste were not assessed (Fetouh, 2015; Changsawake et al., 2017; Teerarak et al., 2020).

### 3.5 Nanotechnology

Nanotechnology has recently been applied as postharvest treatments to prolong the storage period of sweet basil. Nanotechnology has gained traction for its beneficial uses in postharvest preservation and food processing (Neme et al., 2021). Thyme volatile oil loaded chitosan nanoparticles were applied to sweet basil leaves as edible coatings and lengthened shelf life at 20°C by 2.4-fold (Hassan et al., 2021). The nanoparticles minimized fresh weight loss, reduced malondialdehyde and hydrogen peroxide and retained higher levels of chlorophyll, carotenoids and volatile oils, potentially due to delays in decay induction, reduction in ROS and lipid peroxidation, protective effects to the plasma membranes and elevation of antioxidant enzymes. Nano-curcumin and nano-rosmarinic acid were also applied as foliar treatments to harvested plants and were reported to reduce fresh weight loss, slow the degradation of chlorophyll, retain volatile oil content and inhibit microorganism growth at concentrations of 60-90 µg after storage in polyethylene bags for 3 weeks at 10°C, however statistical significance, visual and sensory effects were not described (Hammam and Shoala, 2020).

## 4 Production methods

### 4.1 Artificial lighting

Incorporating green light in indoor growing systems may be more significant in alleviating postharvest cold injury of basil than blue or ultraviolet (UV) light. Red light and blue light generally dominate indoor growing systems due to the ability of plant chlorophylls to efficiently absorb photons in this spectra for photosynthesis and the declining cost of light-emitting diode (LED) lights (Ouzounis et al., 2015). These wavelengths are also important for stimulating the production of secondary metabolites, regulating growth and inducing stomatal openings. Although green light and UV-A light are not as readily absorbed, green light plays a key role in distributing energy throughout the leaves, assimilating CO_2_, increasing biomass and signaling environmental acclimation while UV-A light assists in producing secondary metabolites (i.e., pigments and flavonoids) in certain genotypes and regulating biomass accumulation and morphology (Smith et al., 2017; Verdaguer et al., 2017). Basil grown under different ratios of red (600-700 nm), blue (415-480 nm) and green (480-600 nm) light, with and without supplementation of UV-A (380-415 nm) light, were assessed for shelf life during storage at 20°C for 12 h, 6°C for 12 h and then 20°C for 60 h (Jensen et al., 2018). Red light supplemented with green light (80:20) resulted in basil with a slightly longer shelf life and higher chilling tolerance than the red and blue light combinations with or without UV-A light due to higher water retention. This is supported by the relationship between green light, stomatal function and leaf water retention. Increasing ratios of blue light had either no effect or negatively impacted chilling tolerance and storage performance (Jensen et al., 2018; Larsen et al., 2022a).

Supplementing the growth cycle with far-red light may encourage postharvest cold tolerance in basil. Introducing far-red light to indoor growing systems has gained interest because it regulates photosynthetic capacity, fosters plant height and expands leaf area (Tan et al., 2022). In basil, the addition of far-red light increased plant height and the accumulation of sesquiterpenoids and other volatiles, including 1,8-cineole, that contribute to the complexity and quality of basil aroma (Carvalho et al., 2016; Patel et al., 2021). Basil cultivated indoors under red-white light (400-800 nm at 150 μmol·m^-2^·s^-1^) at 25°C was supplemented with far-red light (700-800 nm at 180 μmol·m^-2^·s^-1^) during the last 0-3 weeks of the 31 day growth cycle (Larsen et al., 2020; Larsen et al., 2023). The longest period of exposure increased plant height, leaf area, percentage of plant dry matter content, plant fresh mass and chilling tolerance in leaves stored at 4°C and 12°C in the dark for 15 days. Visual quality of leaves improved from a score of ~40% to ~60% after 9-12 days of storage at both temperatures. The addition of far-red light had no effect on the hormones (jasmonic acid and abscisic acid) or antioxidants (rosmarinic, chicoric and ascorbic acid), but it did produce more soluble sugars and starch, which likely contributes to the heightened chilling tolerance.

Increasing light intensity and possibly adding UV-B light for the days leading up to harvest enhances the postharvest quality of basil. Higher light intensity and UV-B light are known to intensify the rate of photosynthesis in a variety of plant species, given the intensity remains below saturation levels (Escobar-Bravo et al., 2017; Wimalasekera, 2019). Basil plants grown in a vertical farm were subjected to 5 days of “End-of-Production” (EOP) red-white light (400-800 nm) treatments of 50, 150, 300 or 600 μmol·m^−2^·s^−1^ (Larsen et al., 2020; Larsen et al., 2022b). The higher EOP light intensity grew more resilient leaves, increased plant fresh mass, boosted the percentage of plant dry matter content and slightly elevated the overall visual quality after being stored for 6-12 days in the dark at 4°C and 12°C. These changes were supported by higher levels of soluble sugars, starch and nonenzymatic antioxidants, such as rosmarinic acid and ascorbic acid in the samples receiving more intense light (Larsen et al., 2022b). Despite these correlations, higher intensity EOP light was not reported to alleviate chilling symptoms. A parallel study was conducted with EOP treatments of blue light (400-500 nm) in place of red-white light (Larsen et al., 2022a; Larsen et al., 2023). Intensifying the percentage of blue light increased plant height when the wavelength was 100% blue but had minimal effect on antioxidant production and chilling tolerance for green and purple leaf basil. Yet, blue EOP light with the higher photosynthetic photon flux density (PPFD) of 300 μmol·m^−2^·s^−1^ retained higher levels of soluble sugars and antioxidant content and improved the leaf visual quality after storage in 4°C for 3-9 days and in 12°C for 6-12 days (Larsen et al., 2022a). Furthermore, after exposure to supplemental UV-B light (290-320 nm) at 0.5 kg·s^−3^ for 4 days and then white light for 1 day prior to harvest, fresh basil leaves stored for 7 days in the dark at 10°C showed higher phenolic content and antioxidant activity but no improvement in general visual quality (Dos et al., 2020).

### 4.2 Photoperiod and temperature

The role that photoperiod and growing temperature have on postharvest basil quality remains unclear. Relationships between photoperiod, temperature and chilling injury have been studied extensively in *Arabidopsis.* The expression of most cold-inducible genes peaks in the afternoon before temperatures descend at night, including for the well-known cis-regulatory elements (*CRE* 1-3) and C-repeat binding factors (*CBF* 1-3), while the expression of those typically downregulated by cold peaks around dawn before temperatures rise with the day (Grundy et al., 2015). The circadian clock not only enables plants to acclimate to daily temperature fluctuations, but also to seasonal changes through mechanisms such as alternative splicing and transcription factor regulation. Basil is significantly influenced by the photoperiod. When supplemental fluorescent lighting (125 fc) was used to extend the natural daylight period of 9 h, basil grown under a 24 h light period yielded the greatest biomass but matured for harvest 3-21 days later than those grown under 9-21 h of light (Skrubis and Markakis, 1976). Although 24 h daily illumination increases biomass and induces secondary metabolites associated with flavor, it also causes stunting, chlorosis, lignified stem tissue, leaf necrosis and unusual colorations in basil (Skrubis and Markakis, 1976; Beaman et al., 2009; Johnson et al., 2019). Supplementing outdoor tunnel production of basil with incandescent lamps reduced preharvest chilling injury, possibly due to a rise in the local ambient temperature from 16.1°C to 17.8°C (Dudai et al., 2017). However, when basil plants were grown indoors under red-white light at 15°C and 25°C in the last 3 weeks of harvest, no difference in postharvest chilling tolerance was observed (Larsen et al., 2023). Although not reported, growing at 15°C was likely detrimental to yield and quality as basil grows optimally at temperatures of ~29°C (Currey, 2020). More research is needed to determine how the circadian rhythm, growing temperature and photoperiod influence chilling response in basil.

### 4.3 Drought and salt stress

Water deficit during production largely reduces volatile compounds without impacting the visual quality of stored basil. Growing plants under drought conditions is a strategy used to augment the production of aroma compounds and other secondary metabolites and reduce water use, but potentially at the cost of yield as demonstrated in peppermint (*Mentha* x *piperita* L.), lemongrass (*Cymbopogon* spp.) and thyme (*Thymus* spp.) (Charles et al., 1990; Singh-Sangwan et al., 1994; Ashrafi et al., 2018). The effects of water deficit on basil aroma compounds are complex. For example, some studies have shown that water deficit increases linalool, a key compound in basil driving sweet and floral aroma (Simon et al., 1992; Radácsi et al., 2010), while others have demonstrated water deficit decreases linalool (Khalid, 2006; Jordan et al., 2017). This relationship is further complicated by storage and the different ways in which water deficit is defined and applied (acute vs. chronic, application method, stage of development, etc.). Overall, water stress depleted fresh volatile profiles in basil after 7 days of storage at 12°C under a 12 h light period for Genovese basil, but not during storage for Green Iranian basil (Jordan et al., 2017). The Purple Iranian basil was very sensitive to storage conditions and all treatments were depleted after 5-7 days. While some compounds increased in Genovese basil over storage such as β-pinene and limonene, other key aroma compounds such as linalool decreased significantly, or increased but then decreased after 5 days of storage as in the case of α-pinene, β-pinene, (*Z*)-β-pinene, camphor, chavicol, eugenol and 1,8-cineole. In contrast, storage strengthened phenolic acid content in samples grown at a deficit irrigation of 75% and 50% field capacity compared to the control irrigated at 100% field capacity (Luna et al., 2015). Increasing the production of phenols is beneficial for their role in taste, metabolism and stress, however, the advantages do not offset the negative impacts to aroma profile, which is key for consumer acceptance (Shafran et al., 2007; Luna et al., 2015; Jordan et al., 2017; Patel et al., 2021). Water deficit irrigation can still be a useful strategy to conserve water if needed because water deficit as low as 25% field capacity did not affect visual appearance of basil during storage (Bekhradi et al., 2015).

Mild salt stress during production may improve postharvest quality, but at the cost of lower yield and injury. Many agricultural areas bear high levels of salt accumulation and are unable to grow salt sensitive crops (Grieve et al., 2012). Although moderately tolerant crops can endure environmental salt accumulation, the stress will likely impact their quality and yield. Salt stress commonly reduces the size of fruits and vegetables and thus yield, but can simultaneously amplify the production of secondary metabolites. For instance, a saline nutrient solution (4 dS·m^−1^) supplied to cauliflower plants at each irrigation enhanced the quality and shelf life of cauliflower by enriching the dry matter, soluble content and nutritional profile compared to the control (2 dS·m^−1^) (Giuffrida et al., 2018). In basil, salt stress positively impacted the visual quality of Green Iranian basil by decreasing leaf blackening and maintaining the limit of marketability after 7 days of storage when 40 and 80 mM NaCl were added to the nutrient solution for 25 days during cultivation (Bekhradi et al., 2016). These results were not replicable in Purple Iranian basil or Genovese basil. Additionally, the salt stress reduced leaf thickness in Green Iranian and Genovese basil while lessening transpiration rates, diminishing chlorophyll content and escalating lipid peroxidation in Genovese basil. Yield decreased with salt stress by 0.2-0.8 kg·m^−2^ in Green Iranian basil, 0.3 kg·m^−2^ in Purple Iranian basil and 0.3-0.6 kg·m^−2^ in Genovese basil.

### 4.4. Plant nutrition

Plant nutrition *via* nutrient amendments and biofortification affect the chemical composition of plants and thus impact storage duration. Ideal concentrations of fertilizers and nutrients vary by genotype, cropping system and environment (Teliban et al., 2022). Wide ranges of recommendations exist for the application rates of nitrogen (N), phosphorous (P) and potassium (K) and are 104-200 kg·ha^−1^, 12-100 kg·ha^−1^ and 73-120 kg·ha^−1^, respectively for basil (Sharafzadeh and Alizadeh, 2011). Purdue University extension recommends an NPK ratio of 1-1-1 by broadcast and plow down application of N-P_2_O_5_-K_2_O at a rate of 120-120-120 lbs per acre (Simon, 1985). Other research found that an NPK of 160-80-80 kg·ha^−1^ in combination with farm yard manure (10 t·ha^−1^) yielded the most fresh material and essential oil at time of harvest for basil (Suryanarayana, 1970). Yet, high levels of nitrogen can negatively affect the accumulation of phenolic compounds and flavonoids (Mahlangu et al., 2021). Applications with lower concentrations of nitrogen at 60 kg·ha^−1^ throughout production were optimal for preservation of phenols as this concentration maintained high levels of antioxidant and free radical scavenging activity throughout storage up to 15 days at 12°C, but likely at a reduced yield. Supplementation of nutrient solution with 4 mg·L^−1^ selenium (Se) may also enhance postharvest quality as this concentration reduced ethylene production and produced more antioxidants, total phenols and rosmarinic acid in basil after 5 days of storage in the dark at 8°C (Puccinelli et al., 2020). However, Se biofortification decreased the net photosynthesis rate and elevated nitrate levels in basil, which can be toxic if high enough. Similarly, increasing the concentration of a nutrient solution from single to double strength raised nitrates and nitrites in basil leaves (De Pascale et al., 2006). Silicon amendments may also benefit basil postharvest by alleviating cold stress. Silicon (Si; 75 ppm) incorporated into the nutrient solution of a hydroponic growing system significantly increased basil shoot height and weight without negatively affecting plant morphology and led to higher survival rates with reduced cold injury after an unintentional frost event (Li et al., 2020). How nutrient applications during production affect volatile composition and browning of basil leaves needs to be further studied to understand ideal recommendations for optimizing postharvest quality.

### 4.5 Time of harvest

Harvesting produce later in the afternoon or evening significantly extends postharvest shelf life of basil and other vegetables. Harvesting sweet basil at 18:00 prolonged shelf life nearly two-fold at 10°C and 20°C compared to harvests at 2:00 or 6:00 (Lange and Cameron, 1994). Sweet basil harvested at 18:00 resulted in the longest shelf life at 12-15°C followed by 22:00, 10:00-14:00, 6:00 and 2:00. Similar results have been documented in other crops. Shifting the time of harvest to the end of the day lengthened postharvest shelf life of salad roquette or arugula (*Eruca vesicaria* ssp. *sativa*) by 2-6 days and Lollo Rosso lettuce (*Lactuca sativa* L. ‘Ravita’) and red chard (*Beta vulgaris* L. var. *flavescens*) by 1-2 days (Clarkson et al., 2005). Harvesting basil in the afternoon minimized response to low temperatures during storage. Symptoms of chilling injury were much more severe after storage at 12°C in samples harvested at 6:00 in the morning compared to those harvested at 13:00 in the afternoon as demonstrated in **Figure 3** (Aharoni et al., 2010). Harvesting time had no effect on volatile composition when samples harvested at 8:00 were compared to those harvested at 16:00 after storage at 10°C (da Silva et al., 2005). Season and location can significantly impact the yield and composition of basil essential oil. Harvesting in the summer in Brazil compared to the winter resulted in an overall increase of essential oils at time of harvest and after storage for *O. basilicum* (da Silva et al., 2005). Meanwhile, harvesting in the winter resulted in the highest essential oil concentration at time of harvest in Pakistan for *O. basilicum*, which was richer in oxygenated monoterpenes (Hussain et al., 2008) and in India for *O. gratissimum*, which was richer in limonene and methyl eugenol (Kumar and Lal, 2022). Thus, seasonal and local conditions play a role in producing basil with more aroma to preserve during storage.

## 5 Conclusion

Maximizing environmental and physiological conditions is essential for basil to acclimate to and withstand storage conditions as a cold sensitive crop. Storing and shipping basil at low temperatures is important for reducing the growth of molds, yeasts and bacteria, however lower temperatures deplete aroma and damage the leaves. Storage temperatures of 12-15°C at >90% RH are ideal in addition to the adoption of mitigation strategies, i.e., applying pre- and postharvest treatments, proper storage conditions and best practices for cultivation as shown in **Figure 2** and **Table 1**. The most effective strategies for extending the shelf life of basil include acclimating plants to 10°C for 4 h the day prior to harvest, harvesting plants in the afternoon or evening, treating harvested material with 1-MCP (0.4 g·m^−3^ applied for 8 h) and sanitizers (2% lactic acid and 2% H_2_O_2_ heated to 40°C or 6% NaClO solution), maintaining atmospheric conditions of 1.5-2% O_2_ and 0% CO_2_ in N_2_ during storage and supplementing the last three weeks of the growth cycle (10 DAS) with far-red light (180 μmol·m^-2^·s^-1^). Packaging basil leaves in low density polyethylene bags, storing leaves under partial illumination, applying mild heat treatments of 38-42°C to plants prior to or after harvest, growing plants under higher light intensity, adding 20% green light to indoor growing systems and enhancing harvested material with edible thyme, curcumin and rosmarinic acid nanoparticles have also been shown to improve basil postharvest quality. Other promising strategies that require further research include preharvest treatments with phytohormones such as ABA, postharvest nitric oxide fumigation, supplementing the last few days of the growth cycle with high intensity light, delivering white light pulses, red light pulses or UV-B light during storage, supplementing the production cycle with Se or Si, applying mild salt stress and modifying nitrogen levels in nutrient applications. Future work should seek to validate and continue to improve these techniques not only for storage conditions, treatments and production methods, but also for handling during transportation and shipping to optimize the shelf life of sweet basil.

## Author contributions

LB prepared and edited the original manuscript as this was part of her dissertation studies. JS provided advice and guidance in its preparation and reviewed and edited the manuscript. All authors contributed to the article and approved the submitted version.
